# Self-rated health and its association with perceived environmental hazards, the social environment, and cultural stressors in an environmental justice population

**DOI:** 10.1186/s12889-018-5797-7

**Published:** 2018-08-03

**Authors:** Judy Y. Ou, Junenette L. Peters, Jonathan I. Levy, Roseann Bongiovanni, Alina Rossini, Madeleine K. Scammell

**Affiliations:** 1 Boston University School of Public Health, Boston, MA USA; 2 Huntsman Cancer Institute at the University of Utah, Salt Lake City, UT USA; 3 GreenRoots Inc, Chelsea, MA 02150 USA

## Abstract

**Background:**

Communities with large minority populations often are located near sources of pollution and have higher crime rates, which may work in combination with other factors to influence health. Poor self-rated health is related to chronic health conditions and premature mortality, with minority populations most likely to report poor health. To address how both resident perception of neighborhood environments and chronic health conditions individually and collectively influence health, we examined self-rated health and its association with multiple types of perceived environmental hazards in a majority-Hispanic urban population.

**Methods:**

We conducted interviews with 354 residents of Chelsea, Massachusetts, US and asked about self-rated health, perceptions of their neighborhood, including participant-reported environmental hazards (e.g., air quality, odors and noise), aspects of the social environment (e.g., feeling safe, neighborhood crime, social cohesion), and culture-related stressors (e.g., immigration status, language stress, ethnic identity). Log-linear models examined the independent and multivariable associations between these factors and fair/poor self-rated health, controlling for socio-demographic characteristics and preexisting health conditions.

**Results:**

Forty-one percent of participants reported fair/poor self-rated health. Participants frequently perceived environmental hazards such as problems with pests and regular noise disturbance as well as feeling unsafe. In a multivariable model, a greater number of reported noise disturbances (≥ 2 noise sources = 1.53 [1.04–2.26]) and reported insecurity with immigration status (1.66 [1.01–2.73]) were positively associated with fair/poor self-rated health. High social cohesion was inversely associated (0.74 [0.48–1.14]) with fair/poor self-rated health in the multivariable model.

**Conclusions:**

Negative perceptions of environmental hazards and reported cultural stressors were significantly associated with fair/poor self-rated health among residents in a low-income majority-minority community, with social cohesion having a beneficial association with self-rated health. Efforts to improve health should recognize the importance of public perceptions of social and environmental hazards found in neighborhood environments, and benefits of strengthening community connections.

**Electronic supplementary material:**

The online version of this article (10.1186/s12889-018-5797-7) contains supplementary material, which is available to authorized users.

## Background

Self-rated health is a reflection of a person’s psychological, social, and physical health that a single measure of morbidity cannot measure [1]. Because of the close ties between self-rated health and this larger picture of health, poor self-rated health is strongly correlated with premature mortality [1]. Because self-rated health encompasses the physical and emotional aspects of health itself, understanding how multiple social, physical health, and environmental factors influence self-rated health can provide a better picture of how to improve health ratings and by extension, reduce premature mortality.

Minority populations in the United States (US) systematically report poor self-rated health more frequently than White-Caucasians [2, 3]. This disparity could be attributed to the multiple social, health-related, and environmental problems that cause stress among minority populations. Low socioeconomic status [4], lack of English language proficiency [5], the prevalence of chronic health conditions [6], and social factors (e.g. racial discrimination) [7] have been identified as reasons for the reporting of poor health among minority populations. Minority populations are also more likely to live in neighborhoods with hazardous waste sites and point sources of pollution which may cause health problems and induce feelings of stress, which could also lower health ratings [8, 9]. Rather than examining social and environmental predictors of self-rated health individually, evaluating the combined or aggregate impacts of the physical and social environment alongside chronic health conditions that affect minorities can provide a more complete picture of how these complex sources of stress influence health ratings and thus longevity.

Neighborhoods that feel unsafe, are heavily polluted, or have hostile or isolating social or cultural environments have been linked to poor self-rated health in previous studies [10–12]. The conditions of the neighborhood itself, such as cracked sidewalks and poor lighting, could create feelings of an unwelcoming or stressful environment. These same neighborhood conditions are associated with poor self-rated health and higher mortality [13].

The social environment can moderate or exacerbate the effects of environmental pollution and poor neighborhood conditions [14]. A previous assessment of neighborhood conditions and perceptions of safety found that a lack of green space, feeling unsafe, and problems with litter are associated with poorer self-rated health even after adjusting for income and education [11]. Social cohesion, which describes the feelings of trust and inclusion in social settings, is correlated with good self-rated health, and reduces stress induced by neighborhoods with poor conditions [14, 15]. This moderating effect implies that environmental pollution, social isolation or inclusion, and culture-related stressors, such as low language proficiency [5], may also work in combination to influence health ratings in a negative manner.

We explore the independent and multivariable associations of participant-reported neighborhood environmental hazards, the social environment, and cultural stressors on self-rated health among residents of Chelsea, Massachusetts [16]. Every census tract in Chelsea meets at least one of the four criteria for defining environmental justice communities in Massachusetts: ≥25% percent minority population, ≥25% percent foreign born, annual median household income ≤65% of the statewide median, or ≥25% households lacking English language proficiency [17]. According to the US Census, Chelsea is comprised of a 62% Hispanic population, and 21% of the population is living below the federal poverty level [16]. The city contains several environmental hazards due to the presence of oil storage facilities, a multi-level highway with trucks and heavy traffic, and a designated port area within the city [18]. Chelsea also has one of the highest crime rates in the state of Massachusetts; in 2012, Chelsea’s violent crime rate was nearly five times Massachusetts’ rate [19]. This study is an opportunity to examine how resident perceptions of environmental and social factors are associated with the self-reported health of a majority Hispanic/Latino population.

## Methods

The Chelsea STAR (Science To Achieve Results) project was a community-university partnership to investigate the health and environmental concerns of residents in the previously described environmental justice community through a cross-sectional study [20]. Researchers and community members developed an interview guide containing 180 open- and closed-ended questions, including pre-validated and original questions that specifically address local issues (Additional file 1) [21].

Recruitment occurred between December 2011 and June 2013 via door knocking between 9 am and 8 pm on weekdays and weekends. Cable television channels and flyers posted at community centers, clinics, homes, and local events publicized the study. Eligibility criteria included being 18 years of age or older, residence in the city for six months or more, the ability to speak English or Spanish, and current residence in one of five census tracts located in the city. Interviews were conducted at participants’ homes, or at the office of a community organization downtown. Geographic coordinates of participants’ homes were recorded. Prior to each interview, participants were given a consent letter with information about their participation in the study. Consent was indicated by the participant moving forward with the interview. We included a list of all interview questions relevant to our analyses in the Additional file 1. The Boston University Medical Campus Institutional Review Board approved this study.

We characterized neighborhood environmental hazards as perceptions of environmental pollution, pests, noise and odor disturbances, and neighborhood conditions; the social environment as feeling unsafe, crime, drug use and loitering, and social cohesion; and cultural stressors as ethnic group identity, ethnic group orientation, stress from language, and insecurity with immigration status. Since the reporting of self-rated health may be biased due to linguistic differences or translational effects, we control for language in our analyses [22]. We also include mental and chronic health conditions in the analyses.

### Self-rated health

We measured self-rated health by asking participants to rate their own health, with the response options of excellent, very good, good, fair, or poor. Very few participants reported poor health (*n* = 31, 8.8%), so we dichotomized responses into two categories [23, 24]: 1) excellent/very good/good self-rated health, consisting of the responses excellent, very good, or good (reference), and 2) fair/poor self-rated health, including the responses fair or poor. This dichotomization strategy also has similar results to the original scale [23, 24]. We refer to the outcome as fair/poor self-rated health.

### Health conditions

We assessed chronic and mental health conditions through participant-reported diagnoses of a variety of conditions, including heart disease, diabetes, depression, and anxiety disorders [25]. We developed two variables, a count of chronic health conditions (0, 1, 2, or ≥ 3 conditions) and a dichotomous variable indicating one or more mental health conditions (yes or no). A third variable indicated whether participants had a disability (yes or no). Disability was reported by participants in two ways: as a direct question about employment and as a chronic health condition.

### Neighborhood environmental hazards

We asked residents about their perceptions of air quality, pest problems, noise and odor disturbances, and neighborhood conditions. Perceived air quality was grouped into the categories very good/good (reference), very bad/bad, and uncertain/never thought about it. Participants indicated if rats, mice or insects bothered them in their homes within the past year with a yes or no response [26].

Noise disturbances were assessed as: 1) the number of noise sources that regularly bothered participants (No noise, 1, ≥2 noises); 2) loss of sleep because of noise disturbances (No noise, No sleep disruption, Sleep disruption); and 3) negative emotions were elicited by noise (No negative response, Negative response, No noise). The categorization of the number of noises (No noise, 1, or ≥ 2) was chosen based on the distribution of the data as only 12% of participants reported 3 or more noises.

Odor disturbances were assessed with three similar variables: 1) the number of smells or odors tNhat regularly bothered participants; 2) whether odors from outdoor sources prevented participants from opening their windows or going outside; and 3) if the odors produced a negative physical response (e.g. nausea, headache).

Residents also answered questions from a modified version of the Neighborhood/Block Conditions Assessment tool. This survey was previously used to gather resident perceptions of the social and physical neighborhood environment, such as crime and feeling unsafe, via telephone [27]. The survey was later adapted for use by the Chelsea STAR project [27, 28]. We added 7 questions to the original 13-question instrument based on input from residents and a Chicago-based community survey [29]. From this resulting 20-item instrument, we analyzed seven items to arrive at a measure of poor neighborhood conditions that assessed property damage, poor lighting, graffiti, etc. (Additional file 1).

### Social environment

We defined social cohesion as feelings of inclusion and sense of belonging. Measures of social cohesion identified the extent to which individuals experience trusting relationships, cooperation and participation in their communities [15]. We assessed individual perceptions of neighborhood social cohesion using items from Sampson’s collective efficacy scale [30]. Responses to the four social cohesion survey items were recorded on a Likert scale, where participants indicated their level of agreement or disagreement (scale of 1–4) or neutrality about the statements describing social relationships in their neighborhood. The scale was scored such that participants with higher scores had higher levels of social cohesion.

Feeling unsafe, and concerns about crime, drug use and loitering were measured using the remaining 13 of the 20 items from the modified Neighborhood/Block Conditions Assessment instrument (Additional file 1) [25].

### Cultural stressors

We used an abbreviated version of Phinney’s Multi-group Ethnic Identity Measure to create measures of ethnic identity and other ethnic group orientation, which are separate concepts in Phinney’s instrument [31]. Ethnic identity describes the extent to which individuals participate in social expressions of their culture and ethnicity. Other ethnic group orientation describes interest, comfort and practice of engaging with groups outside of one’s own ethnicity [31]. The two are not mutually exclusive.

We also asked participants if they feel secure with their immigration status. While a small handful of US-born participants said they feel insecure with their immigration status, we only counted foreign-born, non-US citizens that feel insecure with their immigration status among those with immigration status insecure, compared with all US-citizens and immigrants who feel secure.

Finally, we asked if language was a source of stress for participants. We created two categories: All participants who reported that language was not a source of stress, and participants who conducted the interview in Spanish and who reported that language is a source of stress (Reported stress).

### Statistical methods

The 20 questions from the modified CDC Neighborhood/Block Assessment tool described above contained several questions that appeared to address components of larger constructs, but these larger constructs were not previously identified for the survey instrument. For example, several questions in the assessment tool measured participant opinions of neighborhood conditions or feeling unsafe (Additional file 1), but they were not organized in the original survey in way that would make those groups clear. To identify logical groupings for these questions, we used factor analysis with an orthogonal varimax rotation and a loading factor of 0.3 to examine logical groupings [27, 28], and to verify that questions we expected would measure each type of neighborhood problem would fall into those groupings. We anticipated that each of these 20 questions would belong to one of four groups: poor neighborhood conditions, feeling unsafe, neighborhood crime, and drug use and loitering. The factor analysis confirmed these groupings and produced a latent factor score measuring the contribution of each question to the group. We flagged variables with a rotated factor pattern of 0.5 or greater. Using these criteria, we identified four distinct constructs, with one question that overlapped two constructs: poor neighborhood conditions (7 questions), feeling unsafe (4 questions), neighborhood crime (5 questions), and drug use and loitering (5 questions). These groups are also mentioned above in the methods section and Additional file 1. The factor analysis confirmed these groupings.

To reflect the differences in each question’s contribution to the whole construct, we calculated weights for each item in the construct based on each question’s individual score. These weights created a continuous scale with a mean of zero which represented the average score in the original scoring system. Values in this new scoring system are the difference between an individual’s weighted score and the mean of zero, with positive scores indicating more severe problems. We analyzed these items continuously in regression models.

The questions for ethnic identity, other ethnic group orientation, and social cohesion came from survey instruments that previously identified which questions measured each respective construct. However, we weighted questions measuring each construct and scaled the means as we did for the modified Neighborhood/Block Assessment tool. Positive scores indicated high social cohesion, strong ethnic identity, and strong other ethnic group orientation. These scores were divided into tertiles (low, average, high) in the regression models.

We examined the individual and multivariable associations of the self-reported environmental hazards, characteristics of the social environment, and cultural stressors with fair/poor self-rated health. We considered the following variables for inclusion as predictors of fair/poor self-rated health and potential confounders: education as a proxy for socioeconomic status, interview language, age, sex, disability, current smoking status, alcohol consumption within the past month, and chronic and mental health conditions. Of note, as interview language closely tracks with ethnicity (78% who reported Hispanic or Latino ethnicity chose Spanish as their interview language, 99% of participants who were not of Hispanic or Latino ethnicity chose English as their interview language), we did not include both in the model simultaneously. Similarly, we did not control for language in analyses with cultural stressors in the final analysis due to the close relationship between the two variables. Current smoking and alcohol consumption were not included in the final models because they were not associated with the outcome. We also tested for effect modification between the multiple environmental and social factors by education and interview language by stratifying the models by education or interview language and using an interaction term. We also investigated effect modification between the reported environmental hazards (e.g. noise, perceived air quality), social, and cultural stressors using interaction terms in multiple models.

Because of the high prevalence of the outcome, we used robust log-linear regression models with a Poisson distribution to avoid biased effect estimates and confidence levels [32, 33]. To develop the multivariable models, we compared results from the individual models adjusted for sex, education, age, health conditions, language, and disability status with output from stepwise and LASSO selection methods [34]. Variables that were significant in individual models but not in the multivariable model were not included in the final model. We evaluated the potential for over-fitting the model by entering the control variables individually to assess their impact on the effect estimates and confidence intervals. Analyses were performed using SAS version 9.3. Sensitivity analyses for all models that include smoking and alcohol consumption are included in the Additional file 2.

## Results

### Descriptive results

We interviewed 354 participants, the majority of whom are female (68%), Hispanic or Latino (61%), not working (64%), and high school graduates (66%) (Table 1). Approximately 27% of the study population reported a disability that prevents employment, 70% reported at least one chronic health condition, and 37% reported at least one mental health condition. Forty-one percent of participants (144/354) reported fair/poor self-rated health. Of participants who reported fair/poor self-rated health, 85% (123/144) had at least one chronic health condition.

**Table 1:** Study population characteristics

	Median (Range)
Age, years	49 (18–93)
	N (% total)
Sex	
Female	239 (68)
Education	
≥ High school	231 (66)
Ethnicity	
Hispanic or Latino	215 (61)
Interview language^a^	
English	184 (52)
Immigration status	
Feels secure	298 (84)
Feels insecure	56 (16)
Not working	224 (64)
Permanent or temporary disability	96 (27)
Self-rated health	
Good/Very Good/Excellent	207 (58)
Fair/Poor	144 (41)
Chronic health conditions^b^	
No conditions	110 (29)
1 condition	101 (29)
2 conditions	78 (22)
≥ 3 conditions	65 (18)
Mental health conditions^c^	
No conditions	222 (63)
≥ 1 condition	130 (37)
Not current smoker	289 (82)
No alcoholic drinks in past month	194 (56)

^a^Interviews were conducted in English or Spanish

^b^Heart disease, current asthma, diabetes, hypertension, cancer, psoriasis, vitiligo, emphysema or other respiratory disease, arthritis, other self-reported chronic conditions

^c^Depression, anxiety, insomnia, other self-reported mental conditions

Environmental hazards were frequently reported by the participants. Fifty-two percent of participants reported problems with pests, and nearly 50% reported at least one regular noise disturbance (Table 2). Sleep disruption or other negative responses to noise disturbances were noted as problems by 33% of participants. Trucks and planes were the most commonly reported transportation sources of noise.

**Table 2:** Frequency of perceived environmental hazards

		N (%)
Noise disturbance	≥2 noises	89 (25)
	1 noise	79 (22)
	No noise	184 (52)
Negative response to noise	No negative response	38 (11)
	Negative response	110 (31)
	No noise	184 (52)
Sleep disruption from noise	No sleep disruption	50 (14)
	Sleep disruption	118 (33)
	No noise	184 (52)
Odor	Reported odor	123 (35)
	No odor	228 (64)
Odors with negative response	Negative response	74 (21)
	No negative response	36 (10)
	No odor	228 (64)
	Missing	16 (5)
Odors affecting behavior	Cannot open window/go outside	59 (17)
	Can open window/go outside	15 (4)
	No odor	228 (64)
	Missing	52 (15)
Perceived air quality	Very bad/Bad	118 (33)
	Uncertain/Never thought about it	133 (38)
	Very good/Good	98 (28)
Pest problems	Pests	184 (52)
	No pests	165 (47)
Total		354

### Individual model results

Of the array of stressors reported, only noise disturbances from two or more sources (OR = 1.54 [95% CI = 1.22–1.94]) and sleep disruption from noise (1.29 [1.02–1.63]) were positively and significantly associated with fair/poor self-rated health (Table 3) after adjustment for age, sex, education, all health conditions, language, and disability. The worst neighborhood conditions had a positive association with fair/poor self-rated health (1.27 [0.85–1.90], Ref = Best conditions). High and average social cohesion had an inverse association with fair/poor self-rated health (High: 0.80 [0.52–1.23], Average: 0.88 [0.58, 1.31], Ref = Low). Of the cultural stressors, participants who reported feeling insecure with their immigration status were more likely to report fair/poor self-rated health (1.53 [1.12–2.10]). Language-related stress had a non-significant association with the outcome (language stress: 0.94 [0.72–1.22]). These results are similar in the models that included smoking and alcohol consumption (Additional file 2: Table S1).

**Table 3:** Adjusted odds ratios (OR) and 95% CI for the separate models of reported environmental hazards, the social environment, and cultural stressors on fair/poor self-rated health

		Model N	OR	95% CI
Reported environmental hazards^a^				
Noise disturbance	≥ 2 noises	350	1.54**	1.22, 1.94
	1 noise		0.85	0.60, 1.21
	No noise		Ref	
Negative response to noise	No negative response	350	1.27	0.92, 1.74
	Negative response		1.26	0.97, 1.63
	No noise		Ref	
Sleep disruption from noise	No sleep disruption	350	0.91	0.56, 1.47
	Sleep disruption		1.29**	1.02, 1.63
	No noise		Ref	
Odor	≥1 odor	350	1.01	0.79, 1.29
	No odor		Ref	
Odor with negative response	Negative response	329	1.08	0.76, 1.54
	No negative response		0.89	0.65, 1.23
	No odor		Ref	
Odors affecting behavior	Cannot open window/go outside	350	0.96	0.67, 1.39
	Can open window/go outside		0.89	0.34, 2.35
	No odor		Ref	
Perceived air quality	Very bad/Bad	347	1.21	0.90, 1.64
	Uncertain/Never thought about it		1.26	0.94, 1.68
	Very good/Good		Ref	
Pests	Pests reported	347	1.16	0.91, 1.48
	No pests		Ref	
Neighborhood conditions	Worst conditions	350	1.27	0.85, 1.90
	Average conditions		0.94	0.61, 1.45
	Best conditions		Ref	
Social environment^a^				
High social cohesion	Highest cohesion	350	0.80	0.52, 1.23
	Average cohesion		0.88	0.58, 1.31
	Least cohesion		Ref	
Feeling unsafe	Feels least safe	348	1.10	0.71, 1.69
	Feels average safe		1.06	0.68, 1.65
	Feels most safe		Ref	
Perceived crime	Most crime	349	1.12	0.73, 1.72
	Average crime		1.05	0.68, 1.62
	Least crime		Ref	
Drug use and loitering	Most problems with drugs	348	1.07	0.70, 1.63
	Average problems with drugs		0.90	0.58, 1.38
	Least problems with drugs		Ref	
Cultural stressors^b^				
Immigration status	Feels insecure	350	1.53**	1.12, 2.10
	Feels secure		Ref	
Language stress	Reported stress	349	0.94	0.72, 1.22
	No stress		Ref	
Ethnic identity	Identifies strongly with own group	350	0.97	0.65, 1.46
	Identifies with own group		0.97	0.65, 1.45
	Does not identify with own group		Ref	
Ethnic group orientation	Strongly identifies with other groups	350	0.62**	0.39, 0.98
	Identifies with other groups		0.93	0.64, 1.37
	Does not identify with other groups		Ref	

** *p* < 0.05, * *p* < 0.10

^a^Adjusted for age, sex, education, all health conditions, language, disability

^b^Adjusted for age, sex, education, all health conditions, disability

### Multivariable model results

The multivariable model indicated that fair/poor self-rated health had positive associations with reports of two or more noise disturbances (1.53 [1.04–2.26], No noise = ref); interviewing in Spanish (1.49 [1.00–2.23]); and reporting insecure feelings about their immigration status (1.66 [1.01–2.73]) (Table 4). High and average social cohesion had an inverse association with fair/poor self-rated health (High: 0.74 [0.48–1.14], Average: 0.82 [0.54–1.24], Ref = Low). In addition, of the other covariates, fair/poor self-rated health was significantly more prevalent with less than a high school education and a greater number of chronic health conditions. Sleep disturbance from noise was not significant after including noise disturbances in the final model and was not included. These results are also similar to the smoking and alcohol consumption-adjusted models (Additional file 2: Table S2). Models examining effect modification of the environmental and social factors by education, interview language, or other stressors did not yield consistent or statistically significant estimates.

**Table 4:** Odds ratios (OR) and 95% CI for the multivariable model of reported environmental hazards, the social environment, and cultural stressors on fair/poor self-rated health

		OR	95% CI
Environmental hazards			
Noise disturbance	≥2 noises	1.53**	1.04, 2.26
	1 noise	0.79	0.49, 1.29
	No noise	Ref	
Social environment			
High social cohesion	Highest cohesion	0.74	0.48, 1.14
	Average cohesion	0.82	0.54, 1.24
	Least cohesion	Ref	
Cultural stressors			
Immigration status	Feels insecure	1.66**	1.01, 2.73
	Feels secure	Ref	
Population characteristics			
Interview language	Spanish	1.49*	1.00, 2.23
	English	Ref	
Age	45–59 years	1.11	0.73, 1.68
	> 60 years	0.92	0.55, 1.54
	18–44 years	Ref	
Education	<High school	1.49**	1.03, 2.17
	≥High school	Ref	
Sex	Female	1.12	0.75, 1.69
	Male	Ref	
Chronic health conditions	1 condition	1.96**	1.12, 3.46
	2 conditions	2.88**	1.60, 5.16
	≥3 conditions	2.98**	1.59, 5.70
	No conditions	Ref	
Mental health conditions	≥1 condition	1.12	0.75, 1.68
	No conditions	Ref	
Disability	Reported disability	1.40	0.94, 2.10
	No disability	Ref	

**p < 0.05, *p < 0.10; Model *N* = 350

## Discussion

Our findings support the importance of addressing environmental hazards, the social environment, and cultural stressors when studying predictors of self-rated health. Although addressing the burden of chronic health conditions and disability is important to improve the health of minority communities, our analyses suggest that the role of the physical and social environments on self-rated health should be recognized as significant contributing factors separate from preexisting chronic physical or mental illness. We report that participant-reported noise disturbances, social cohesion, and insecurity with respect to immigration status are associated with fair/poor self-rated health even after considering the influence of chronic health conditions, disability, and language. These environmental influences are especially relevant in Chelsea, which is largely Hispanic/Latino, low-income, and located in the flight path of and directly adjacent to an international airport and under a large multilevel highway.

The association between participant-reported noises and self-rated health could have multiple explanations. Noise can induce cardiovascular changes that increase risk for mortality [35], cardiovascular disease outcomes [35–37], and can serve as a proxy measure for a variety of environmental hazards, such as air pollution. Participants reported noise sources that originate from sources of pollution (e.g. trucks), so greater number of reported noise disturbances could indicate more exposure to environmental contaminants. Annoyance from traffic and sleep disruption due to noise are identified as mediators on the pathway from noise to fair/poor self-rated health in previous literature [38, 39]. Noise is also associated with a higher frequency of reported annoyance, anxiety and depression among residents [40, 41], and are risk factors for subsequent chronic health conditions [42]. This study cannot confirm whether participants who reported more noises are exposed to more pollution, so the association between noise and self-rated health could represent multiple factors.

The inverse correlation between social cohesion and fair/poor self-rated health could be due to the role of social support in reducing stress [43]. The robustness of the inverse association in the multivariable model provides evidence for the protective effect of social cohesion noted in previous studies [14, 43]. Previous studies note that the moderating effect is only present if problems with environmental conditions are mild to moderate [14, 43]. While we cannot formally compare participant-reported environmental conditions in our study with measures elsewhere and were underpowered to address complex interactions, we note that the estimates for high and average social cohesion are not attenuated when included in the multivariable model with noise and physical health conditions.

Insecurity with immigration status is a major barrier to social inclusion and improving health. Nearly 20% of our study population reports stress due to immigration status that significantly affects their health rating in an adverse manner. The association between insecurity with immigration status and fair/poor self-rated health could be driven by fear of deportation or social isolation. Both documented and undocumented Latino immigrants in the US report fear of deportation, which may prevent Latino immigrants from obtaining employment and health care services [44]. Latino immigrants in the US also report stress due to separation from friends and family, and isolation from the general community due to social and linguistic barriers [45, 46]. In our study, Spanish interview language is correlated with fair/poor self-rated health, which could be attributed to the effect of language or translation [5]. Immigration status is currently a complex and sensitive topic in the US, and understanding how insecurity with immigration status affects health of minority populations is a topic of great importance requiring additional research.

### Strengths and limitations

Limitations include the cross-sectional study design, which does not allow us to determine causality or control for social selection (sick people move into one specific neighborhood) or social causation (neighborhoods cause illness) [47]. Because we rely on perceived measures of the environment rather than direct objective measurements, our associations may be due to differential reporting of environmental hazards based on the current health of participants [48]. It is possible that people with more illnesses are more likely to report fair/poor self-rated health and worse environmental conditions. Including chronic health conditions and mental health in our analysis helps control for this dependent, differential misclassification. Although language is a proxy measure for ethnicity and is correlated with ethnicity, we did not include a direct measure of race or ethnicity in the analysis. We also did not have information on household income, so we were unable to control for income in the analysis. We also lack the sample size to examine the individual items on the self-rated health question in a multinomial regression model. However, dichotomization of the outcome is a validated method with similar results to the original question [23, 24].

Despite these limitations, we identify strong associations among fair/poor self-rated health and participant-reported environmental hazards, the social environment, and cultural stressors.

## Conclusion

This is one of the few studies on self-rated health that incorporates sources of cultural and social stress with environmental hazards found in neighborhood environments. Furthermore, we confirm the roles of environmental, social, and cultural stressors on self-rated health even after adjusting for chronic and mental health conditions. Our findings shed light on the complex sources of chronic stress in an environmental justice community, and their impacts on the health of a majority-Latino population. In addition to focusing on the treatment of clinical disease, alleviating the burden of stress from environmental hazards, the social environment, and cultural stressors found in neighborhoods is one method to improve self-rated health.

## Additional files

Additional file 1: Interview Questions. (DOCX 41 kb)

Additional file 2: **Table S1.** Sensitivity analysis including smoking and alcoholic drink consumption as predictors for the separate models of reported environmental hazards, the social environment, and cultural stressors on fair/poor self-rated health. **Table S2.** Sensitivity analysis including smoking and alcoholic drink consumption as predictors for the multivariable model of environmental hazards, the social environment, and cultural stressors on fair/poor self-rated health. (DOCX 37 kb)
