# Supplementary material for: Self-rated health and its association with perceived environmental hazards, the social environment, and cultural stressors in an environmental justice population
Source: BMC Public Health. 2018 Aug 3;18:970. doi: 10.1186/s12889-018-5797-7 (PMC6090753; doi:10.1186/s12889-018-5797-7)
Supplement: Supplementary file 1 — Interview Questions. (DOCX 41 kb) [file 12889_2018_5797_MOESM1_ESM.docx]

Additional file 1: INTERVIEW QUESTIONS

Table of Contents

[Self-Rated Health 2](#_Toc510540651)

[Environmental Hazards 2](#_Toc510540652)

[Social Environment 6](#_Toc510540653)

[Cultural Stressors 9](#_Toc510540654)

[Health Conditions 10](#_Toc510540655)

[Factor Analysis Results 14](#_Toc510540656)

# Self-Rated Health

Would you say that in general your health is:

1□ Excellent 2□ Very good 3□ Good 4□ Fair 5□ Poor 7□ No response

# Environmental Hazards

*Perceived Air Quality*

Generally speaking, what do you think about the air quality in Chelsea?

Choose from these responses [Refer to response options card]:

| 1□ Very bad |
| --- |
| 2□ Bad |
| 3□ Good |
| 4□ Very good |
| 5□ I have never thought about it |
| 6□ I am uncertain |
| 7□ Refused |

*Pests* (Shalowitz et al. 1998)

The following questions ask about events in your life that may have contributed to feeling stress. You may answer Yes or No to each question.

In the last year…

Did rats, mice or insects bother you in your home? 1□ Yes 0□ No

*Noise Count, Negative Response, and Sleep Disturbance*

When at home, are there noises that regularly bother you? 0□ No 1□ Yes

*[If yes,]* Please, can you tell me what noises bother you?

[For each source of noise checked on list ask the following questions]:

How does *[fill in noise]* make you feel?

Do you ever lose sleep because of *[fill in noise]*?

|  | Noise | Feeling | Sleep | |
| --- | --- | --- | --- | --- |
| 1□ | Street traffic |  | 0□ No 1□ Yes |  |
| 1□ | Trucks |  | 0□ No 1□ Yes |  |
| 1□ | Noisy neighbors |  | 0□ No 1□ Yes |  |
| 1□ | People talking or shouting in the street |  | 0□ No 1□ Yes |  |
| 1□ | Jets, airplanes, helicopters |  | 0□ No 1□ Yes |  |
| 1□ | Trains |  | 0□ No 1□ Yes |  |
| 1□ | Emergency vehicle sirens |  | 0□ No 1□ Yes |  |
| 1□ | Car alarms |  | 0□ No 1□ Yes |  |
| 1□ | Cars playing loud music |  | 0□ No 1□ Yes |  |
| 1□ | Loud music |  | 0□ No 1□ Yes | |
| 1□ | Other: _______________ |  | 0□ No 1□ Yes | |
| 1□ | Refused |  | 0□ No 1□ Yes | |

*Odor Count, Negative Response, and Behavioral Effect*

When at home, are there smells or odors that ever bother you? 0□ No 1□ Yes

*[If yes,]* Please, can you tell me what smells or odors bother you?

[For each source of smell checked on list ask the following questions]:

How does *[fill in odor]* affect you or make you feel?

Does the *[fill in smell/odor]* occur at any particular time of day, or in any pattern that you are aware of?

Does the *[fill in smell/odor]* ever keep you from going outside or opening your windows?

|  | Odor | Feeling | Time | Impact |
| --- | --- | --- | --- | --- |
| 1□ | Petroleum or oil |  |  | 0□ No 1□ Yes |
| 1□ | Low tide |  |  | 0□ No 1□ Yes |
| 1□ | Vehicle emissions or exhaust |  |  | 0□ No 1□ Yes |
| 1□ | Sewer |  |  | 0□ No 1□ Yes |
| 1□ | Kayem hot dog factory |  |  | 0□ No 1□ Yes |
| 1□ | Boston Hides and Furs / company that works with animal skins |  |  | 0□ No 1□ Yes |
| 1□ | Other: ___________________ |  |  | 0□ No 1□ Yes |
| 1□ | Refused |  |  | 0□ No 1□ Yes |

*Poor Neighborhood Conditions* (Ou et al. 2015)

The next set of questions is about the conditions of your neighborhood. I will ask you about something people may think is a problem in your neighborhood, and you respond with either (0) No opinion, (0) No problem, (1) Minor problem, (2) Serious problem.

| Property damage? | 0□ No problem/  No opinion | 1□ Minor problem | 2□ Serious problem |
| --- | --- | --- | --- |
| Poor lighting on the streets at night? | 0□ No problem/  No opinion | 1□ Minor problem | 2□ Serious problem |
| Graffiti on buildings and walls? | 0□ No problem/  No opinion | 1□ Minor problem | 2□ Serious problem |
| Irregular trash pickup? | 0□ No problem/  No opinion | 1□ Minor problem | 2□ Serious problem |
| Poor city services? | 0□ No problem/  No opinion | 1□ Minor problem | 2□ Serious problem |
| Litter, trash or broken glass on sidewalks? | 0□ No problem/  No opinion | 1□ Minor problem | 2□ Serious problem |
| Vacant or abandoned houses, storefronts and lots? | 0□ No problem/  No opinion | 1□ Minor problem | 2□ Serious problem |

# Social Environment

*Social Cohesion* (Sampson et al. 1997)

Now I will read four statements and you choose the response that best represents your agreement about what I said. The options are: (1) Strongly agree, (2) Agree, (3) Neither agree nor disagree, (4) Disagree, (5) Strongly disagree. These statements are about your neighborhood and the people who live here.

| People are willing to help their neighbors. | | |  |  |
| --- | --- | --- | --- | --- |
| 1□ Strongly agree | 2□ Agree | 3□ Neither agree nor disagree | 4□ Disagree | 5□ Strongly disagree |
| This is a close-knit neighborhood | | |  |  |
| 1□ Strongly agree | 2□ Agree | 3□ Neither agree nor disagree | 4□ Disagree | 5□ Strongly disagree |
| People in this neighborhood can be trusted. | | |  |  |
| 1□ Strongly agree | 2□ Agree | 3□ Neither agree nor disagree | 4□ Disagree | 5□ Strongly disagree |
| People in this neighborhood don’t get along with each other. | | | | |
| 1□ Strongly agree | 2□ Agree | 3□ Neither agree nor disagree | 4□ Disagree | 5□ Strongly disagree |
| Responses for the last question were reverse coded. | | |  |  |

*Feeling Unsafe* (Ou et al. 2015)

The next set of questions is about the conditions of your neighborhood. I will ask you about something people may think is a problem in your neighborhood, and you respond with either (0) No opinion, (0) No problem, (1) Minor problem, (2) Serious problem.

| Feeling unsafe in your home? | 0□ No problem/  No opinion | 1□ Minor problem | 2□ Serious problem |
| --- | --- | --- | --- |
| Feeling unsafe while out alone on the street during the day? | 0□ No problem/  No opinion | 1□ Minor problem | 2□ Serious problem |
| Feeling unsafe alone during the night? | 0□ No problem/  No opinion | 1□ Minor problem | 2□ Serious problem |
| Slow police response or police protection? | 0□ No problem/  No opinion | 1□ Minor problem | 2□ Serious problem |

*Neighborhood Crime* (Ou et al. 2014)

The next set of questions is about the conditions of your neighborhood. I will ask you about something people may think is a problem in your neighborhood, and you respond with either (0) No opinion, (0) No problem, (1) Minor problem, (2) Serious problem.

| Gangs? | 0□ No problem/  No opinion | 1□ Minor problem | 2□ Serious problem |
| --- | --- | --- | --- |
| Gunshots? | 0□ No problem/  No opinion | 1□ Minor problem | 2□ Serious problem |
| Physical fighting? | 0□ No problem/  No opinion | 1□ Minor problem | 2□ Serious problem |
| Physical assaults of people on the street? | 0□ No problem/  No opinion | 1□ Minor problem | 2□ Serious problem |
| Prostitution? | 0□ No problem/  No opinion | 1□ Minor problem | 2□ Serious problem |

*Drug Use and Loitering* Ou et al. 2015)

The next set of questions is about the conditions of your neighborhood. I will ask you about something people may think is a problem in your neighborhood, and you respond with either (0) No opinion, (0) No problem, (1) Minor problem, (2) Serious problem.

| Drug dealing and/or use? | 0□ No problem/  No opinion | 1□ Minor problem | 2□ Serious problem |
| --- | --- | --- | --- |
| Group of people hanging around with nothing to do? | 0□ No problem/  No opinion | 1□ Minor problem | 2□ Serious problem |
| No or few supervised activities for youth? | 0□ No problem/  No opinion | 1□ Minor problem | 2□ Serious problem |
| Too few recreational facilities available for young people? | 0□ No problem/  No opinion | 1□ Minor problem | 2□ Serious problem |
| Prostitution? | 0□ No problem/  No opinion | 1□ Minor problem | 2□ Serious problem |

# Cultural Stressors

*Ethnic Identity* (Phinney 1992)

For the following questions I will make a statement and ask you to say how well the statement represents your own thoughts. You may say you: Strongly agree, Agree, No opinion, Disagree, or Strongly disagree.

| I am active in organizations or social groups that include mostly members of my own ethnic group | | | | |
| --- | --- | --- | --- | --- |
| 1□ Strongly agree | 2□ Agree | 3□ Neither agree  nor disagree | 4□ Disagree | 5□ Strongly disagree |
| I participate in cultural practices of my own group, such as special food, music, or customs. | | | | |
| 1□ Strongly agree | 2□ Agree | 3□ Neither agree  nor disagree | 4□ Disagree | 5□ Strongly disagree |
| I have a clear sense of my ethnic background and what it means to me. | | | |  |
| 1□ Strongly agree | 2□ Agree | 3□ Neither agree  nor disagree | 4□ Disagree | 5□ Strongly disagree |

*Ethnic Group Orientation* (Phinney 1992)

For the following questions I will make a statement and ask you to say how well the statement represents your own thoughts. You may say you: Strongly agree, Agree, No opinion, Disagree, or Strongly disagree.

| I like meeting and getting to know people from ethnic groups other than my own. | | | | |
| --- | --- | --- | --- | --- |
| 1□ Strongly agree | 2□ Agree | 3□ Neither agree  nor disagree | 4□ Disagree | 5□ Strongly disagree |
| I sometimes feel it would be better if different ethnic groups did not try to mix together. | | | | |
| 1□ Strongly agree | 2□ Agree | 3□ Neither agree  nor disagree | 4□ Disagree | 5□ Strongly disagree |
| I often spend time with people from ethnic groups other than my own. | | | |  |
| 1□ Strongly agree | 2□ Agree | 3□ Neither agree  nor disagree | 4□ Disagree | 5□ Strongly disagree |
| I am involved in activities with people from other ethnic groups. | | |  |  |
| 1□ Strongly agree | 2□ Agree | 3□ Neither agree nor disagree | 4□ Disagree | 5□ Strongly disagree |

Age: What year were you born?

*Feeling Insecure with Immigration Status*

Do you feel secure about your immigration status? 0□ No 1□ Yes 7□ No response

Are you a citizen of the United States? 0□ No 1□ Yes 7□ No response

Do you consider yourself an immigrant? 0□ No 1□ Yes 7□ No response

*Language Stress*

Is language ever a source of stress for you? 0□ No 1□ Yes 7□ No response

# Health Conditions

*Chronic Health Conditions*

Diabetes (Center for Disease Control and Prevention 2010)

Have you ever been told by a doctor that you have diabetes?

[If "Yes" and respondent is female, ask: "Was this only when you were pregnant?"]

| 1□ Yes |
| --- |
| 2□ Yes, but female told only during pregnancy |
| 3□ No |
| 4□ Pre-diabetes or borderline diabetes |
| 7□ No response/Don’t know/Not sure |

Cardiovascular Disease (Center for Disease Control and Prevention 2010)

Has a doctor, nurse, or other health professional EVER told you that you

had any of the following?

(Ever told) a heart attack, also called a myocardial infarction?

0□ No 1□ Yes 7□ No response

(Ever told) angina or coronary heart disease? 0□ No 1□ Yes 7□ No response

(Ever told) a stroke? 0□ No 1□ Yes 7□ No response

Asthma or Respiratory Disease (Center for Disease Control and Prevention 2010)

Have you EVER been told by a doctor, nurse, or other health professional that you had asthma? 0□ No 1□ Yes 7□ No response

*[If yes]* Do you still have asthma? 0□ No 1□ Yes

Has a doctor or other health professional EVER told you that you had emphysema or some other respiratory disease? 0□ No 1□ Yes 2□ Emphysema 3□ Other ________

7□ No response

Arthritis (Center for Disease Control and Prevention 2010)

Have you EVER been told by a doctor or other health professional that you have some form of arthritis, rheumatoid arthritis, gout, lupus, or fibromyalgia? 0□ No 1□ Yes 7□ No response

Hypertension (Center for Disease Control and Prevention 2010)

Have you EVER been told that you had hypertension (hy-per-ten-shun), also called high blood pressure?

[Interviewer instruction: If person says "high normal blood pressure", "borderline hypertension" or "prehypertension" code "No" below].

[If "Yes" and respondent is female, ask: "Was this only when you were pregnant?"]

1□ Yes

2□ Yes, but female told only during pregnancy

0□ No [Go to next section]

7□ Don’t know/Not sure

9□ No response

Skin Conditions (Center for Disease Control and Prevention 2010)

Psoriasis (sore-eye-asis)? [if asked, a chronic skin condition that appears like an itchy rash] 0□ No 1□ Yes 7□ No response

Vitiligo (Vit-i-LI-go)? [if asked, a condition that results in loss of pigment in patches of skin] 0□ No 1□ Yes 7□ No response

Cancer (Center for Disease Control and Prevention 2010)

Have you ever been told by a doctor that you had a type of cancer or malignant tumor?

0□ No 1□ Yes

*[If Yes]* Where in the body, or what organ, did the cancer or tumor start?

­______________________________________________________________

Other Chronic Health Conditions

Do you have any other chronic medical condition? 0□ No 1□ Yes

*[If Yes]* Please specify what condition:

_____________________________________________________________

*Mental Health Conditions*

Depressive Disorders (Center for Disease Control and Prevention 2010)

Has a doctor or other healthcare provider EVER told you that you have a depressive disorder (including depression, major depression, or minor depression)?

0□ No 1□ Yes 7□ No response

Other mental health conditions

Do you have any other chronic medical condition? 0□ No 1□ Yes

*[If Yes]* Please specify what condition: _________

# Factor Analysis Results

| **Rotated Factor Pattern** | **Factor 1:**  **Neighborhood Crime** | **Factor 2:**  **Poor Neighborhood Conditions** | **Factor 3:**  **Feeling Unsafe** | **Factor 4:**  **Drug use and loitering** |
| --- | --- | --- | --- | --- |
| Physical assaults of people on the street? | 69* | 10 | 37 | 11 |
| Gangs? | 73* | 20 | 16 | 11 |
| Physical fighting? | 73* | 20 | 38 | 18 |
| Gunshots? | 67* | 23 | 23 | 23 |
| Prostitution? | 58* | 17 | -5 | 41† |
| Property damage? | 27 | 57* | 13 | 16 |
| Poor lighting on the streets at night? | 21 | 53* | 46 | -10 |
| Vacant or abandoned houses, storefronts and lots? | 21 | 66* | 7 | -2 |
| Graffiti on buildings and walls? | 26 | 69* | 8 | 17 |
| Poor city services? | 9 | 58* | 25 | 29 |
| Litter, trash or broken glass on sidewalks? | 4 | 55* | 15 | 39 |
| Irregular trash pickup? | -8 | 39† | 24 | 28 |
| Feeling unsafe while out alone on the street during the day? | 18 | 10 | 64* | 35 |
| Feeling unsafe alone during the night? | 32 | 16 | 66* | 27 |
| Feeling unsafe in your home? | 15 | 22 | 75* | 8 |
| Slow police response or police protection? | 23 | 25 | 47† | 22 |
| Drug dealing and/or use? | 43 | 21 | -3 | 61* |
| Group of people hanging around with nothing to do? | 41 | 15 | 32 | 56* |
| No or few supervised activities for youth? | 21 | 13 | 37 | 62* |
| Too few recreational facilities available for young people? | 15 | 20 | 28 | 63* |
| Printed values are multiplied by 100 and rounded to the nearest integer. Values greater than 0.5 are flagged by an '*'. Values flagged by an “†” were included in the factor even through the score was less than 0.5 | | | | |

References

Center for Disease Control and Prevention. 2010. Behavioral risk factor surveillance system. Available: <http://www.cdc.gov/brfss/questionnaires.htm> [accessed 2010.

Ou, JY, Carlin C, Olortegui RM, Peters JL, Bongiovanni R, Scammell MK. Chelsea STAR Annotated Interview Guide. http://www.ces4health.info. 2015

Phinney JS. 1992. The multigroup ethnic identity measure: A new scale for use with diverse groups. Journal of Adolescent Research 7:156–176.

Sampson RJ, Raudenbush SW, Earls F. 1997. Neighborhoods and violent crime: A multilevel study of collective efficacy. Science 277:918–924.

Shalowitz M, Berry C, Rasinski K, Dannhausen-Brun C. 1998. A new measure of contemporary life stress: Development, validation, and reliability of the crisys. Health Serv Res Dec 1998:22.
