# Supplementary material for: Self-rated health and its association with perceived environmental hazards, the social environment, and cultural stressors in an environmental justice population
Source: BMC Public Health. 2018 Aug 3;18:970. doi: 10.1186/s12889-018-5797-7 (PMC6090753; doi:10.1186/s12889-018-5797-7)
Supplement: Supplementary file 2 — Table S1. Sensitivity analysis including smoking and alcoholic drink consumption as predictors for the separate models of reported environmental hazards, the social environment, and cultural stressors on fair/poor self-rated health. Table S2. Sensitivity analysis including smoking and alcoholic drink consumption as predictors for the multivariable model of environmental hazards, the social environment, and cultural stressors on fair/poor self-rated health. (DOCX 37 kb) [file 12889_2018_5797_MOESM2_ESM.docx]

**Supplemental Tables**

**Table of Contents**

[Supplemental Table 1. Sensitivity analysis including smoking and alcoholic drink consumption as predictors for the separate models of reported environmental hazards, the social environment, and cultural stressors on fair/poor self-rated health 1](#_Toc511928739)

[Supplemental Table 2. Sensitivity analysis including smoking and alcoholic drink consumption as predictors for the multivariable model of environmental hazards, the social environment, and cultural stressors on fair/poor self-rated health 3](#_Toc511928740)

# Table S1. Sensitivity analysis including smoking and alcoholic drink consumption as predictors for the separate models of reported environmental hazards, the social environment, and cultural stressors on fair/poor self-rated health

|  |  | **OR** | **95% CI** |
| --- | --- | --- | --- |
| **Reported environmental hazards^a^** |  |  |  |
| Noise disturbance | 1 noise | 0.87 | 0.53, 1.41 |
|  | >2 noises | 1.56** | 1.07, 2.30 |
|  | No noise | Ref |  |
| Negative response to noise | No negative response | 0.53 | 0.25, 1.12 |
|  | Negative response | 1.12 | 0.76, 1.64 |
|  | No noise | Ref |  |
| Sleep disruption from noise | No sleep disruption | 0.91 | 0.48, 1.74 |
|  | Sleep disruption | 1.32 | 0.92, 1.90 |
|  | No noise | Ref |  |
| Odor | ≥1 odor | 1.03 | 0.72, 1.48 |
|  | No odor | Ref |  |
| Reported odor causing emotional response | Odor, no negative emotions | 1.08 | 0.62, 1.87 |
|  | Odor with negative emotions | 0.92 | 0.57, 1.47 |
|  | No odor | Ref |  |
| Odors affecting behavior | Affected behavior | 0.98 | 0.59, 1.64 |
|  | Did not affect behavior | 0.96 | 0.30, 3.14 |
|  | No odor | Ref |  |
| Perceived air quality | Bad, Very bad | 1.18 | 0.75, 1.86 |
|  | Uncertain/Haven't thought | 1.22 | 0.78, 1.89 |
|  | Very good, good | Ref |  |
| Pests | Pests reported | 1.18 | 0.82, 1.70 |
|  | No pests | Ref |  |
| Poor neighborhood conditions | Worst conditions | 1.27 | 0.85, 1.90 |
|  | Average conditions | 0.94 | 0.61, 1.45 |
|  | Best conditions | Ref |  |
| **Social environment^a^** |  |  |  |
| High social cohesion | Highest cohesion | 0.80 | 0.52, 1.23 |
|  | Average cohesion | 0.88 | 0.58, 1.32 |
|  | Least cohesion | Ref |  |
| Feeling unsafe | Feels least safe | 1.06 | 0.68, 1.65 |
|  | Feels average safe | 1.10 | 0.71, 1.69 |
|  | Feels most safe | Ref |  |
| Perceived crime | Most crime | 1.12 | 0.73, 1.72 |
|  | Average crime | 1.05 | 0.68, 1.62 |
|  | Least crime | Ref |  |
| Drug use and loitering | Most problems with drugs | 1.07 | 0.70, 1.63 |
|  | Average problems with drugs | 0.90 | 0.58, 1.38 |
|  | Least problems with drugs | Ref |  |
| **Cultural stressors^b^** |  |  |  |
| Immigration status | Feels insecure | 1.95** | 1.01, 3.79 |
|  | Feels secure | Ref |  |
| Language stress | Reported stress | 1.03 | 0.73, 1.45 |
|  | No stress | Ref |  |
| Ethnic identity | Identifies strongly with own group | 1.00 | 0.66, 1.49 |
|  | Identifies with own group | 1.00 | 0.66, 1.51 |
|  | Does not identify with own group | Ref |  |
| Ethnic group orientation | Strongly identifies with other groups | 0.63** | 0.40, 0.99 |
|  | Identifies with other groups | 0.93 | 0.64, 1.37 |
|  | Does not identify with other groups | Ref |  |
| **p<0.05, *p<0.1 | |  |  |
| ^a^Adjusted for age, sex, education, all health conditions, language, disability, current smoking, and alcohol consumption | | | |
| ^b^Adjusted for age, sex, education, all health conditions, disability, current smoking, and alcohol consumption | | | |

# Table S2. Sensitivity analysis including smoking and alcoholic drink consumption as predictors for the multivariable model of environmental hazards, the social environment, and cultural stressors on fair/poor self-rated health

|  |  | **OR** | **95% CI** |
| --- | --- | --- | --- |
| **Environmental hazards** |  |  |  |
| Noise disturbance | 1 noise | 0.80 | 0.49, 1.32 |
|  | ≥2 noises | 1.54** | 1.05, 2.28 |
|  | No noise | Ref |  |
|  |  |  |  |
| **Social environment** |  |  |  |
| High social cohesion | Highest cohesion | 0.74 | 0.48, 1.14 |
|  | Average cohesion | 0.84 | 0.54, 1.25 |
|  | Least cohesion | Ref |  |
| **Cultural stressors** |  |  |  |
| Immigration status | Feels insecure | 1.80 | 0.91, 3.58 |
|  | Feels secure | Ref |  |
|  |  |  |  |
| **Population characteristics** |  |  |  |
| Interview language | Spanish | 1.44 | 0.95, 2.19 |
|  | English | Ref |  |
| Age | 45–59 years | 1.13 | 0.74, 1.71 |
|  | >60 years | 0.93 | 0.56, 1.55 |
|  | 18–44 years | Ref |  |
| Education | <High school | 1.44 | 0.98, 2.11 |
|  | >High school | Ref |  |
| Sex | Female | 1.08 | 0.71, 1.65 |
|  | Male | Ref |  |
| Chronic health conditions | 1 condition | 2.03** | 1.15, 3.59 |
|  | 2 conditions | 2.88** | 1.60, 5.19 |
|  | ≥3 conditions | 3.01** | 1.60, 5.68 |
|  | No conditions | Ref |  |
| Mental health conditions | ≥1 condition | 1.11 | 0.74, 1.66 |
|  | No conditions | Ref |  |
| Disability | Reported disability | 1.39 | 0.93, 2.09 |
|  | No disability | Ref |  |
| Smoking | Current smoker | 0.97 | 0.66, 1.43 |
|  | No smoking | Ref |  |
| Alcohol consumption | ≥1 drink | 0.87 | 0.60, 1.28 |
|  | No drinks | Ref |  |
| ** Significant p<0.05; Model N = 347 | | | |
